# Primary synovial sarcomas of the bone: clinical perspectives and correlation between the application of SS18-SSX and decalcification status

**DOI:** 10.3389/fonc.2023.1265824

**Published:** 2023-12-18

**Authors:** Jiro Ichikawa, Tomonori Kawasaki

**Affiliations:** ^1^ Department of Orthopaedic Surgery, Interdisciplinary Graduate School of Medicine, University of Yamanashi, Chuo, Japan; ^2^ Department of Pathology, Saitama Medical University International Medical Center, Hidaka, Japan

**Keywords:** surgery, bone, chemotherapy, decalcification, FISH, immunohistochemistry, synovial sarcoma

## 1 Introduction

We read with great interest an article titled “Primary synovial sarcoma of bone: a retrospective analysis of 25 patients” published in *Histopathology* (1). In that study, Righi et al. evaluated the diagnostic accuracy of two novel antibodies (2), SS18:SSX fusion-specific antibody (clone E9X9V; cat# 72364) and SSX-specific antibody targeting the SSX C-terminus (clone E5A2C; cat# 23855), in decalcified surgical specimens and the outcomes of synovial sarcoma (SS) derived from bone. They found that, for primary bone SS, SS18::SSX had 92% (23/25) sensitivity and 99% specificity, whereas SSX had 100% (25/25) sensitivity and 94% specificity. However, fluorescence *in situ* hybridization (FISH) analysis was feasible in only 9 (36%) cases, and SS18 rearrangement was detected in all 9. Also, 7 (28%) patients received chemotherapy with methotrexate, cisplatin, doxorubicin, or ifosfamide. A significant improvement in 10-year disease-free survival (DFS) was observed in patients who received chemotherapy compared to those who did not (*P* = 0.039). Their series highlighted the usefulness of SS18::SSX fusion-specific and SSX C-terminal antibodies in supporting SS diagnosis, particularly when a molecular analysis is not feasible. Furthermore, appropriate adjustment of chemotherapy has been associated with improved outcomes (1). We wondered about the decalcification methods, information on FISH and immunohistochemistry (IHC) in each case, details of surgery, and prognosis. We wish to introduce the significance of this study with correspondence from the authors.

## 2 Subsections relevant for the subject and discussion

In recent years, based on advances in cancer genomics, “precision” medicine has been attracting research attention, as well as molecularly targeted therapeutic agents, the importance of proper specimen handling, which includes preparation of formalin-fixed paraffin-embedded (FFPE) blocks and quality control. Regarding the decalcification of hard tissues, the ethylenediaminetetraacetic acid (EDTA) method is recommended when performing IHC and gene analyses, leading to appropriate diagnoses and treatments. Demineralization using acetic acid or hydrochloric acid/formic acid should generally be avoided (3). During the formalin fixation of tissues for IHC, cross-linking reactions can mask the epitope of the target protein and prevent antibody binding to the antigen. Antigen retrieval, which re-exposes epitopes, is mainly divided into heat and proteolytic enzyme methods. Epitope retrieval with heat treatment can be performed using devices such as microwaves, water baths, and autoclaves, and various types and/or pH buffers, such as citrate, EDTA, and Tris-EDTA, are used for heating (4).

In this notable research by Righi et al., pretreatment for antigen retrieval was performed at 95°C with Tris–EDTA, pH8 for 20 min, as stated in the ‘Materials and methods’ section.^1^ However, there is no mention of the decalcification treatments for primary SS of bone, i.e., the process leading up to the fabrication of FFPE blocks. Recently, van Es et al. demonstrated the optimal decalcification of breast cancer bone metastases with EDTA. Without acetic acid or hydrochloric/formic acid, it affects neither IHC nor FISH results for the estrogen receptor, progesterone receptor, or human epidermal growth factor receptor-2 (HER2), an important biomarker for obtaining prognostic information and predicting therapeutic effects (3).

Therefore, in this investigation, we would like to ascertain specifically 1) the type of decalcifying solution used (EDTA, or according to circumstances, hydrochloric, formic, acetic acid), 2) the demineralization time required in each case, 3) the actual situation of “excessive” decalcification in 13 cases for which FISH analysis was non-informative, and 4) the demineralized status of SS18::SSX IHC-negative cases. Clarifying these issues will increase the value of this significant scientific article.

Fortunately, we have successfully corresponded with the authors and received the following unequivocal answers.

1) Our decalcifying protocol involved the use of a homemade solution composed of formic acid (4.25%) and nitric acid (2.6%) that we used for the gross specimen. Meanwhile, for the biopsy or the small sample, we used a commercial weak decalcified solution (Microdecfast^®^). Where possible, a dedicated sample was used for molecular analysis, which was processed without a demineralizing treatment on which any molecular investigations could be performed.

2) All samples with evaluable molecular results (samples 8, 9, 14, 16, 19, 21, 22, 24, and 25) were not treated with decalcifying solutions. In these cases, molecular investigations were performed using a dedicated sample without demineralizing treatment. In all other samples, the demineralization time included a period of 4–6 h of treatment in a homemade decalcifying solution for the oldest sample and a commercial weak decalcified solution (Microdecfast^®^) for the most recent specimen of the tumor obtained from the gross specimen.

3) Patients with uninformative FISH were very old (1970–1999). Excessive decalcification treatment meant that in the samples analyzed with FISH, it was impossible to observe the presence of nuclei in the interphase, indicating an overly aggressive treatment.

4) All decalcified samples for which immunohistochemical investigations were performed were tested for the expression of vimentin as a positive control marker to confirm the immunoreactivity of the samples. All samples in this study that tested negative for SS18::SSX in IHC showed good expression of the control marker vimentin. This demonstrates the reliability of the results. Negative controls were included for each experiment.

The analysis of FISH was limited to “decalcification” and/or “old” samples, even if FISH was performed in a specialist center with good handling. In addition, FISH has not been performed in any hospital because of its high cost and time-consuming processing. Therefore, IHC is valuable for practical applications. The sensitivity and specificity of the SS18::SSX and SSX antibodies in the present study were comparable to those of two previous reports (1, 5). Non-specific staining, sometimes observed in necrotic cells, was almost negative for these two antibodies (5). Great caution is needed regarding false negatives in decalcified samples and false positives in some types of sarcomas (1, 5). IHC with two antibodies will soon become the gold standard for the diagnosis of SS instead of molecular testing, including FISH and RT-PCR.

Despite the retrospective nature of this study and its relatively small sample size, this report clearly facilitates elucidation of the clinical aspects of primary bone SS. Second, we were concerned about selecting operative procedures because amputation was performed in 10 of the 25 patients (40%) (2). Limb salvage has generally been selected for malignant bone neoplasms because local recurrence does not reportedly differ between limb salvage and amputation in osteosarcoma cases (6). Furthermore, limb salvage achieved better 5-year survival and functional outcomes than amputation. Thus, amputation is usually limited to cases in which it is impossible to assure an adequate safety margin and a mega-prosthesis after limb salvage fails; however, the amputation rate was reported to be 27% (6). Based on the literature and our experience, we hope to understand the details of the treatment decision-making process in amputation cases, accompanied by the relevant clinical features and/or imaging findings. Based on the literature and our experience, we hope to understand the details of the treatment decision-making process in amputation cases, accompanied by the relevant clinical features and/or imaging findings. In addition, regarding overall survival (OS), the authors described the 5-year (66.6%) and 10-year OS (47.9%) in bone SS patients as lower than the 5-year OS (79.7%) in soft-part SS patients (7). We asked the authors about the surgery and prognosis and received the following comments:

1) Considering the local treatment decision-making process, we confirmed that a multidisciplinary approach, the gold standard according to current guidelines (8), has been practiced at our institute for many decades. Therefore, a multidisciplinary tumor board discussed each case from the current series, and the treatment approach was decided based on patient- and tumor-related aspects. Survival and possibilities of limb-sparing surgery have gradually improved for patients with osteosarcoma and Ewing’s sarcoma thanks to the introduction of (neo-)adjuvant chemotherapy and modern imaging technologies toward the end of the 20th century. In contrast, the cases included in this series were diagnosed over a very long period. Only 4/25 cases were treated in the last two decades, and all were managed with limb-salvage surgery. Furthermore, only 7 patients with bone synovial sarcoma underwent chemotherapy, and none of those undergoing amputation received neoadjuvant chemotherapy. Finally, 3 patients in the amputation group had tumors in the foot or ankle region, which is a challenging site for limb salvage, and amputation generally offers the best functional outcome.

There is no doubt that adjuvant chemotherapy, in the case of osteosarcoma and Ewing’s sarcoma, contributes to the trend of limb salvage surgery. Even though only 28% with SS of bone in the present study were given chemotherapy, 10 years DFS in the patients with chemotherapy was significantly higher than without chemotherapy. Considering all these observations, we speculate that future prospective investigations may confirm the benefits of combining chemotherapy with limb salvage strategies.

## 3 Conclusion

In this paper, we introduce Righi et al.’s paper. Although 25 cases were small, the paper had high-quality clinical information and pathological findings and could elucidate the nature of SS of bone. SS of bone seemed to change from “extremely rare” to “rare.” The diagnosis of SS using IHC in both soft tissues and bone will become a general trend to prevent misdiagnosis. Further studies are needed to develop a standard therapy of SS of bone SS.

## Author contributions

JI: Conceptualization, Investigation, Writing – original draft, Writing – review & editing. TK: Conceptualization, Investigation, Writing – original draft, Writing – review & editing.

## References

[1] Righi A Gambarotti M Benini S Gibertoni D Asioli S Magagnoli G. Primary synovial sarcoma of bone: A retrospective analysis of 25 patients. Histopathology (2022) 80:686–97. doi: 10.1111/his.14602 34821406

[2] Baranov E McBride MJ Bellizzi AM Ligon AH Fletcher CDM Kadoch C. A novel SS18-SSX fusion-specific antibody for the diagnosis of synovial sarcoma. Am J Surg Pathol (2020) 44:922–33. doi: 10.1097/PAS.0000000000001447 PMC7289668 32141887

[3] van Es SC van der Vegt B Bensch F Gerritse S van Helden EJ Boon E. Decalcification of breast cancer bone metastases with EDTA does not affect ER, PR, and HER2 results. Am J Surg Pathol (2019) 43:1355–60. doi: 10.1097/PAS.0000000000001321 31283631

[4] Pillai G Roberts H Gatter K Pezzella F. p53 expression in normal paraffin-embedded tissue using different antibodies and antigen retrieval buffer systems. Histopathology (2003) 42:83–7. doi: 10.1046/j.1365-2559.2003.01563.x 12493030

[5] Zaborowski M Vargas AC Pulvers J Clarkson A de Guzman D Sioson L. When used together SS18-SSX fusion-specific and SSX C-terminus immunohistochemistry are highly specific and sensitive for the diagnosis of synovial sarcoma and can replace FISH or molecular testing in most cases. Histopathology (2020) 77:588–600. doi: 10.1111/his.14190 32559341

[6] Qi L Ren X Liu Z Li S Zhang W Chen R. Predictors and Survival of Patients with Osteosarcoma after Limb Salvage versus Amputation: A Population-Based Analysis with Propensity Score Matching. World J Surg (2020) 44:2201–10. doi: 10.1007/s00268-020-05471-9 32170370

[7] Hagiwara Y Iwata S Ogura K Kawai A Susa M Morioka H. Clinical analysis of multimodal treatment for localized synovial sarcoma: A multicenter retrospective study. J Orthop Sci (2023) 28:261–6. doi: 10.1016/j.jos.2021.09.012 34756517

[8] Strauss SJ Frezza AM Abecassis N Bajpai J Bauer S Biagini R. Bone sarcomas: ESMO-EURACAN-GENTURIS-ERN PaedCan Clinical Practice Guideline for diagnosis, treatment and follow-up. Ann Oncol (2021) 32:1520–36. doi: 10.1016/j.annonc.2021.08.1995 34500044

